# Elastic Light Scatter Pattern Analysis for the Expedited Detection of *Yersinia* Species in Pork Mince: Proof of Concept

**DOI:** 10.3389/fmicb.2021.641801

**Published:** 2021-02-17

**Authors:** Stephen L. W. On, Yuwei Zhang, Andrew Gehring, Valery Patsekin, Venkata Chelikani, Steve Flint, Haoran Wang, Craig Billington, Graham C. Fletcher, James Lindsay, J. Paul Robinson

**Affiliations:** ^1^Department of Wine, Food and Molecular Biosciences, Lincoln University, Lincoln, New Zealand; ^2^Eastern Regional Research Center, Agricultural Research Service, USDA, Wyndmoor, PA, United States; ^3^Department of Basic Medical Sciences, Purdue University, West Lafayette, IN, United States; ^4^School of Food and Advanced Technology, Massey University, Palmerston North, New Zealand; ^5^Institute of Environmental Science and Research, Christchurch, New Zealand; ^6^New Zealand Institute for Plant & Food Research Limited, Auckland, New Zealand; ^7^Agricultural Research Service, Office of National Programs, USDA, Washington, DC, United States; ^8^Weldon School of Biomedical Engineering, Purdue University, West Lafayette, IN, United States

**Keywords:** *Yersinia*, diagnostics, detection, elastic light scatter, food

## Abstract

Isolation of the pathogens *Yersinia enterocolitica* and *Yersinia pseudotuberculosis* from foods typically rely on slow (10–21 day) “cold enrichment” protocols before confirmed results are obtained. We describe an approach that yields results in 39 h that combines an alternative enrichment method with culture on a non-selective medium, and subsequent identification of suspect colonies using elastic light scatter (ELS) analysis. A prototype database of ELS profiles from five *Yersinia* species and six other bacterial genera found in pork mince was established, and used to compare similar profiles of colonies obtained from enrichment cultures from pork mince samples seeded with representative strains of *Y. enterocolitica* and *Y. pseudotuberculosis.* The presumptive identification by ELS using computerised or visual analyses of 83/90 colonies in these experiments as the target species was confirmed by partial 16S rDNA sequencing. In addition to seeded cultures, our method recovered two naturally occurring *Yersinia* strains. Our results indicate that modified enrichment combined with ELS is a promising new approach for expedited detection of foodborne pathogenic yersiniae.

## Introduction

In industrialised nations, yersiniosis is most frequently associated with infection by either *Yersinia enterocolitica* or *Yersinia pseudotuberculosis* and is typified by sequelae including diarrhoea and acute, severe abdominal pain that may resemble appendicitis (Rosner et al., 2013; Bancerz-Kisiel and Szweda, 2015); fatalities may also occur (ECDC, 2019). These species represent a significant burden of gastrointestinal disease in Europe (ECDC, 2019), New Zealand (Pattis et al., 2019), and the United States (CDC, 2019). Transmission is most commonly associated with the consumption of contaminated food, with pork a frequently mentioned source for *Y. enterocolitica* in particular (Fois et al., 2018; CDC, 2019; ECDC, 2019). However, both *Y. enterocolitica* and *Y. pseudotuberculosis* infections and outbreaks have also been attributed to the consumption of raw or contaminated vegetables such as carrots, spinach and lettuce (MacDonald et al., 2016; Williamson et al., 2016). Both *Yersinia* species have been recovered from a wide range of food, pet, and wild animals (Bancerz-Kisiel and Szweda, 2015; Le Guern et al., 2016; Nousiainen et al., 2016; Joutsen et al., 2017), making epidemiological studies to resolve sources of human infection difficult.

A further obstacle in the rapid response to outbreaks, and source attribution of yersiniosis infections in humans lies in the shortcomings of currently used detection methods. *Yersinia* species are often found in association with other bacterial taxa that share growth properties, may be present in greater numbers and for which selective agents are relatively ineffective (Petsios et al., 2016). The methods most commonly recommended for detection of yersiniae in foods (Weagant et al., 2017) exploits their ability to grow at lower temperatures than competing microflora; however, this “cold enrichment” approach requires a 10–21 day incubation period that is unsuited for rapid response to a suspected outbreak. Clearly better methods for isolation and detection are warranted for improved public health actions.

Elastic light scatter (ELS) analysis involves the examination of individual bacterial colonies on solid media using laser light and the subsequent detection of photons that are scattered after their passage through the colony (Bae et al., 2011, 2012). For many bacteria, the light scatter image is species-specific and this technique has been used to discriminate foodborne pathogens including *Vibrio*, *Campylobacter*, *Listeria*, and *Arcobacter* species (Banada et al., 2007; Huff et al., 2012; He et al., 2015; Patsekin et al., 2019) as well as *Salmonella*, *Escherichia* and *Staphylococcus* species at the genus level (Banada et al., 2009). ELS is a non-destructive technique, allowing bacteria to be further characterised by epidemiological subtyping methods, if required, for outbreak analysis.

This paper describes a prototype approach combining a modified enrichment procedure with ELS analysis to detect *Y. enterocolitica* and *Y. pseudotuberculosis* in pork mince in 39 h.

## Materials and Methods

### Strains

A list of the strains used appears in Table 1. Twenty-two type or reference *Yersinia* isolates were included, spanning five known or potentially foodborne pathogenic species (*Y. enterocolitica*, *Y. pseudotuberculosis*, *Yersinia intermedia*, *Yersinia kristensenii*, and *Yersinia frederiksenii*). A further 18 isolates representing *Enterococcus*, *Aeromonas*, *Macrococcus*, *Morganella, Proteus*, and *Vagococcus* species that were isolated during preliminary isolation experiments on pork mince were also included. After purification, isolates were identified to genus level using BLAST comparisons of partial 16S rRNA sequences.

**TABLE 1:** Strains used.

BEAM class (total no. of ELS colony images included in database)	Species	Strain		Note
*Yersinia enterocolitica (800)*	*Yersinia enterocolitica*	NZ	NZRM 2603	ATCC 9610. (CIP 80-27, DSM 4780). Type strain. Biotype 1b. Serotype O:8. *ail*(–). *virF*(–)
			ERL 053484	Biotype 1A. Avian isolate
			ERL 112277	Biotype 2
			NZRM 767	Biotype 3. Chinchilla, Denmark
			ERL 1084	Biotype 4. Human origin
			ERL 10782	Biotype 4. Human origin
			ERL 032123	Biotype 4. Human origin
			ERL 032124	Biotype 4. Human origin
		United States	ATCC 27729	O8 (biotype 1)
			NCTC 11174	O9 (biotype 2)
			ATCC 49397	Quality control strain for BBL products
*Yersinia pseudotuberculosis (436)*	*Yersinia pseudotuberculosis*	NZ	NZRM 768	ATCC 29833. *ail*(–). *virF*(–). Turkey. Type strain
			ERL 110237	New Zealand outbreak strain (Williamson et al., 2016)
		United States	PB 1+	Serotype O:1b
			ATCC 6903	Serotype O:1b; Maltose negative Schutze’s Group I
*Yersinia* other species (993)	*Yersinia frederiksenii*	NZ	NZRM 2534	Type strain. *ail*(–). *virF*(–). Sewage, Denmark
	*Yersinia kristensenii*	NZ	NZRM 2535	Type strain. *ail*(+). *virF*(–). Human urine
		United States	NRRL B-41454	Ground beef isolate
			ATCC 33639	Hare, United States
	*Yersinia intermedia*	NZ	NZRM 2604	Type strain. *ail*(–). Human urine
		United States	GB1-G1-A1	Ground beef isolate
			NRRL B-41442	Ground beef isolate
*Aeromonas* spp. (708)	*Aeromonas* spp.	NZ	LU1, LU 12, LU20a, LU 19, LU113, LU183, LU47, LU159	Pork mince isolates (this study)
*Enterococcus* spp. (204)	*Enterococcus* spp.	NZ	LU14-7B	Pork mince isolates (this study)
*Macrococcus* spp. (131)	*Macrococcus* spp.	NZ	LU121, LU185	Pork mince isolates (this study)
*Morganella* spp. (337)	*Morganella* spp.	NZ	LU2, LU167	Pork mince isolates (this study)
*Proteus* spp. (142)	*Proteus* spp.	NZ	LU24, LU “swarm”	Pork mince isolates (this study)
*Vagococcus* spp. (216)	*Vagococcus* spp.	NZ	LU116, LU 199	Pork mince isolates (this study)

### Elastic Light Scatter Strain Profile Database Development

Bacterial strains were subcultured twice on 5% blood agar media and grown overnight before a single colony was inoculated into 25 ml nutrient broth (Oxoid, Basingstoke, United Kingdom) and cultured overnight (15–20 h) at 37°C in a shaking incubator set at 100 rpm. Aliquots were taken and serially diluted by 10^–6^ and 10^–7^ in sterile phosphate buffered saline. Tryptone soya agar (TSA) plates (25 ml, “heavy fill”) (Fort Richard, Auckland, NZ) were inoculated with 50μl aliquots from each dilution and inocula evenly distributed over the media using a sterile disposable spreader. Plates were incubated at 28°C for 22 h and then scanned using a custom-built ELS device as described previously (Patsekin et al., 2019). Images of colonies were assimilated into the Baclan software (Purdue University, United States), and databases containing measurements for Zernike moments, Haralick textures, and Patsekin elements of the ELS profiles for taxa established, as described previously (Patsekin et al., 2019). The relative similarity of profiles assigned to the same taxon, and separation of different taxa, was assessed using a cross validation (CV) algorithm (Banada et al., 2009). Satisfactory performance is considered when infrataxonomic values exceed 90 and intertaxonomic values are below 10 (JR, unpublished observations).

### Enrichment Method

Pork mince was purchased from a local supermarket and examined prior to the “best before” date. Samples of 10 g were taken and mixed well into 90 ml of enrichment broth placed together in a classic lab blender bag (Seward, Worthing, United Kingdom) using a stomacher (Bagmixer, Interscience, France). The enrichment broth used was as described previously (Premaratne et al., 2012), modified by adjusting the pH to 8.5 by the addition of laboratory grade 3M potassium hydroxide. The pH of the enrichment broth immediately after the addition of the pork mince was measured, and ranged from 8.1 to 8.3.

Samples were incubated for 37°C under shaking conditions as described above for 17 h, after which time aliquots were diluted to 10^–7^ and 10^–8^ in buffered peptone water (Fort Richard, Auckland, New Zealand), spread-inoculated onto TSA, incubated for 22 h and then examined by ELS as described above. To maximise isolation of non-*Yersinia* species for the identification database, two samples were enriched using unadulterated nutrient broth no. 2 (Oxoid) and processed as above.

To evaluate the efficacy of this protocol for recovery of *Yersinia*, initial experiments were conducted using autoclaved mince to circumvent the influence of naturally occurring competitive microflora. 10 g of autoclaved pork mince was inoculated with dilutions of 24 h cultures of strains ERL 10782 (*Y. enterocolitica)* and ERL 110237 (*Y. pseudotuberculosis)* as low as 10 colony forming units (cfu)/g. Samples were incubated as described above, with bacterial counts conducted on 5% blood agar to promote recovery. Since these experiments were monocultural, ELS was not undertaken. Experiments were performed in triplicate.

### Spiking Experiments, Identification, and Confirmation

A 24 h cultures of *Y. enterocolitica* strain ERL 10782 and *Y. pseudotuberculosis* strain ERL 110237 were used to spike otherwise unadulterated pork mince samples blended with enrichment broth as described above. For *Y. enterocolitica*, in three separate experiments, one colony (≡10^7^ cfu) and five colonies were used as the inocula; in two experiments, the inocula comprised five colonies only. For *Y. pseudotuberculosis*, three (≡10^7^ cfu) and 9–10 colonies were used as the inocula, since these colonies were smaller.

Subsequent experiments to evaluate the limit of detection (LOD) of the enrichment/ELS protocol were conducted using inocula of each of ERL 10782 and ERL 110237 calibrated to deliver as few as 10 cfu/g per unadulterated pork mince sample. Experiments were performed in triplicate.

Spiked mince samples were then incubated overnight and aliquots of the enrichment medium cultured on TSA as described above. Plates were then scanned and colonies imaged as above. ELS profiles for each colony were then compared to databases containing similar profiles for the taxa included, as described above. Up to 10 individual colonies identified as the target organism by the Baclan software using parameters outlined previously to “best fit” these images to the database (Banada et al., 2009) were subcultured; rarely (6/91 colonies examined in all our seeding experiments), the operators visual analysis of ELS profiles was used to identify colonies of interest. The identity of suspect colonies was confirmed by partial (ca. 1,000 bp) 16S rRNA sequence analysis using the F8-27 primer (AGA GTT TGA TCC TGG CTC AG) adapted from Weisburg et al. (1991) and subsequent comparison to the NCBI public database using BLAST (Altschul et al., 1990). Experiments were conducted on different mince samples on each of five different occasions, for each of the *Y. enterocolitica* and *Y. pseudotuberculosis* strains used.

## Results

### Elastic Light Scatter Profile Databases

**FIGURE 1:**
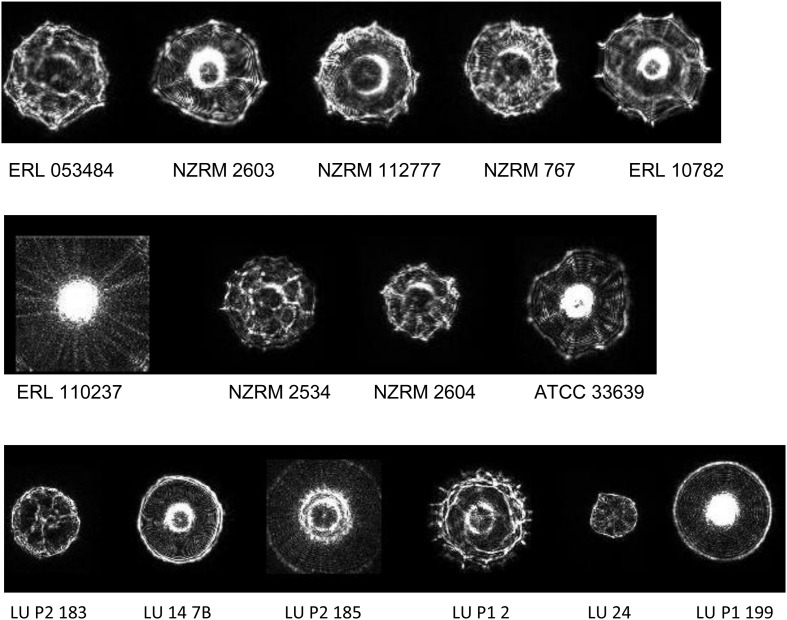
Representative elastic light scatter profiles for colonies of the bacteria examined in this study. Top line: *Y. enterocolitica* strains of Biotype 1a, 1b, 2, 3, and 4 (L-R). Middle line: *Yersinia* spp. *Y. pseudotuberculosis*, *Y. frederiksenii*, *Y. intermedia*, and *Y. kristensenii* (L-R). Bottom line: pork mince strains of *Aeromonas*, *Enterococcus*, *Macrococcus*, *Morganella*, *Proteus*, and *Vagococcus* spp. (L-R). NZRM, New Zealand Reference culture collection (Medical); ATCC, American Type Culture Collection; ERL, Enteric Reference Laboratory, ESR Ltd., LU, Lincoln University.

Representative profiles for the taxa examined are given in Figure 1. Spectra were generally distinctive for the taxa from which they had been derived which correlates with the findings of previous studies using ELS (Banada et al., 2007; Huff et al., 2012; He et al., 2015; Patsekin et al., 2019). However, profiles of *Y. intermedia*, *Y. kristensenii*, and *Y. frederiksenii* were not well separated from each other in the coefficient of variance (CV) matrix (Supplementary Table 1). Therefore, profiles of these species were assimilated into a single group (“*Yersinia* other species”) for the purposes of the identification database. The resulting CV matrix used for identification performed close to the ideal specifications, with results for *Y. pseudotuberculosis* somewhat below the desirable 90 metric (Table 2). Type and reference strains for *Y. enterocolitica* biovars studied (Table 1) were also used to construct a database to assess biovar-level specificity with promising results (Supplementary Table 2). The pathogenic potential of these biovars has been considered to vary, with *Y. enterocolitica* biovar 1A described by some as nonpathogenic (Bancerz-Kisiel et al., 2018). However, pathogenic potential is extant (Batzilla et al., 2011) and human gastrointestinal infections of this taxon in New Zealand show an unusually high incidence (Pattis et al., 2019).

**TABLE 2:** Performance characteristics of the prototype Elastic Light Scatter profile matrix used to identify *Y. enterocolitica* and *Y. pseudotuberculosis* from spiked pork mince.

	*Enterococcus* spp.	*Aeromonas* spp.	*Macrococcus* spp.	*Morganella* spp.	*Proteus* spp.	*Vagococcus* spp.	*Yersinia*, other species	*Y. pseudo- tuberculosis*	*Y. entero- colitica*
*Enterococcus* spp.	100	0	0	0	0	0	0	0	0
*Aeromonas* spp.	0	95.8	0.6	0.9	0.5	0	1.8	0.2	0.7
*Macrococcus* spp.	0	6.1	93.9	0	0	0	0	0	0
*Morganella* spp.	0	3.9	0	85.1	0	0	6.6	0	0.8
Proteus spp.	0	2.8	0.4	0	96.5	0	0.3	0	0
*Vagococcus* spp.	0	0	0.4	0	0	94.5	1.1	4	0
*Yersinia*, other species	0	1.4	0	1	0.1	0.2	91	2.6	3.7
*Y. pseudotuberculosis*	0	0.2	0.4	0.9	0	4	13.2	79.7	0.6
*Y. enterocolitica*	0	0.7	0	0.8	0	0	3.9	0.6	94

*Values given are coefficient of variance figures transformed as described before (Banada et al., 2009). Values close to 100 represent profiles exhibiting high similarity to those under comparison; values of zero indicate profiles are completely dissimilar. Preferred metrics for homologous comparisons exceed 90.*

After assessing the potential of ELS to discriminate yersiniae, a prototype database for use with identifying *Y. enterocolitica* and *Y. pseudotuberculosis* strains in pork mince was developed, comprising ELS profiles from each of the yersiniae studied, as well as database entries for other bacteria recovered from pork mince in preliminary studies. Performance characteristics of this database are given in Table 2 and were considered adequate for challenge studies.

### Efficacy of Enrichment Protocol for Seeded Autoclaved Mince

For *Y. enterocolitica* ERL 10782, initial seedings of as low as 10 cfu/g were recovered to a mean value of 3.9 × 10^7^ cfu/g using the enrichment procedure described. For *Y. pseudotuberculosis* ERL 110237, initial seedings of as low as 10 cfu/g were recovered to a mean value of 1.9 × 10^6^ cfu/g.

### Preliminary Screening of Pork Mince for Other Microflora

Studies were undertaken using our isolation protocol (described above) to evaluate the range of non-yersiniae culturable microorganisms that could be found in pork mince, so that a suitable identification database could be established that minimised false positive identifications. Over several months, 18 isolates representing six different genera (Table 1) were recovered from different batches of pork mince, purified, identified to genus level using 16S rRNA gene comparisons by BLAST and images added to the ELS database alongside those of the *Yersinia* species studied (example images shown in Figure 1). Furthermore, two *Y. enterocolitica* isolates were also recovered during our studies using this protocol.

### Detection and Identification of *Y. enterocolitica* and *Y. pseudotuberculosis* in Spiked Raw Pork Mince Samples

**FIGURE 2:**
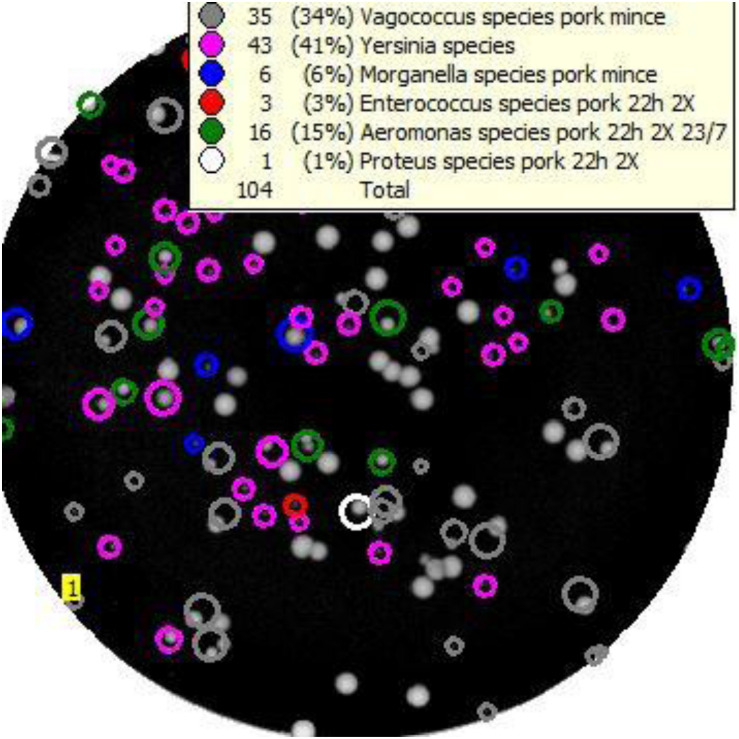
Example of the “Baclan” software output of a TSA medium plate upon which a 10^– 7^ dilution of pork mince enrichment has been cultured and examined by elastic light scatter profiling. Colonies are assigned a presumptive identification for confirmation and/or subtyping as required.

An example of the output from Baclan software where a TSA plate has been scanned and analysed (Figure 2) demonstrates the presumptive identification of individual colonies on the media to taxa contained in the database, based upon their ELS profiles. Using the highest inocula, a total of 27 colonies identified by the software as *Y. enterocolitica* and 23 isolates identified as *Y. pseudotuberculosis* from each of the respective six spiking experiments were subcultured onto TSA for purification, DNA extraction, and molecular identification using BLAST analyses of their partial 16S rDNA sequences. The correct identity was confirmed in 27/27 *Y. enterocolitica* isolates, and 20/23 isolates identified as *Y. pseudotuberculosis.* Incorrectly classified *Y. pseudotuberculosis* isolates were *Aeromonas* (*n* = 2) or *Serratia* spp. Six colonies classified by ELS as *Yersinia* “other species” (see above) were confirmed by BLAST as *Y. enterocolitica* in four independent mince samples seeded with this organism; however, four other colonies classified as *Yersinia* “other species” were not yersiniae, instead representing either *Aeromonas* (*n* = 2) or *E. coli*/*Shigella* spp. (*n* = 2).

Subsequent experiments using inocula of as low as 10 cfu/g for each strain were undertaken to determine the LOD of the enrichment/ELS protocol for raw pork mince samples. For ERL 110237, initial seedings as low as 10^5^ cfu/g could be detected, although this result was only attained in one of three experiments, with recovery consistent using an higher (10^6^ cfu/g) inoculum. Of the nine isolates identified as *Y. pseudotuberculosis* by our custom ELS profile database, all were confirmed as this species by BLAST analysis. Visual analysis of ELS profiles further identified another strain of *Y. pseudotuberculosis* which otherwise would have been misidentified as *Aeromonas* by our database.

Isolates of *Y. enterocolitica* from seeded experiments were more frequently detected and to lower levels. Inocula as low as 10^4^ cfu/g yielded isolates identified by ELS and confirmed by BLAST to this species. Of the 31 isolates recovered and characterised in these experiments, 11 (35.5%) were correctly identified by the ELS database and confirmed as this species, while a further 8 (26%) were identified either as *Y. pseudotuberculosis* (*n* = 4) or “*Yersinia* other species” (*n* = 4) and subsequently confirmed as *Y. enterocolitica.* A further five isolates (16%) were identified by ELS as either *Aeromonas*, *Morganella* or *Enterococcus* spp., but further characterised based on the operators visual analysis. Seven strains (22.5%) were misidentified as “*Yersinia* other species” but represented *Serratia*, *Providentia*, *Pasteurella*, or *Morganella* species, of which the first three genera were not isolated during our initial screening studies and thus not represented in the ELS database.

### Isolation of Naturally Occurring *Y. enterocolitica* Strains

**FIGURE 3:**
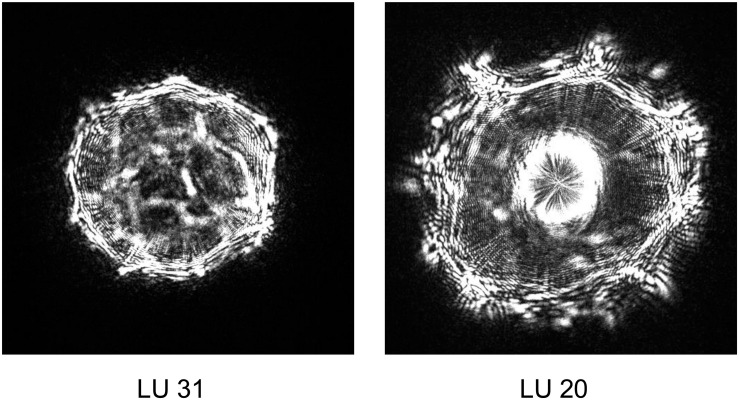
Representative elastic light scatter profiles of naturally occurring *Yersinia* strains recovered in this study.

During our studies, two *Yersinia* strains identified by ELS, yet distinct from that used to seed different pork mince samples, were recovered (Figure 3). Strain LU 31 yielded an ELS phenotype more closely resembling that of *Y. enterocolitica* biotype 1A (cf. Figures 1, 3) with a partial 16S rRNA gene sequence 98.15% similar to a reference strain (NCTC 13769) of this species. The partial 16S rRNA gene sequence of strain LU 20 exhibited the closest (96.48%) similarity to reference strains of *Y. intermedia*, *Y. pekkanenii*, *Y. kristensenii*, *Yersinia aleksicae*, and *Yersinia aldovae.* In contrast, the highest similarity to a reference *Y. enterocolitica* strain (NCTC 13769) was 94.76%. While this does not preclude the strain representing one of *Y. enterocolitica*, it is sufficiently different from results from others using the seeded strain to indicate its novelty. Determination of the genome sequences of both strains is underway.

## Discussion

Current approaches to the detection of *Y. enterocolitica* and *Y. pseudotuberculosis* in foods generally involve enrichment culture to attain a quantum of viable bacterial cells that meet or exceed the LOD of whatever system used, whether conventional or molecular. The challenges in this regard for these species are well established (Premaratne et al., 2012; Petsios et al., 2016; Van Damme et al., 2017; Weagant et al., 2017) and described briefly above. The current protocol recommended by the FDA for detection of *Y. enterocolitica* involves a 10 day cold enrichment step, which for *Y. pseudotuberculosis* extends to 21 days (Weagant et al., 2017). An alternative standardised method ratified for use across Europe (Hallanvuo et al., 2019) demonstrates reasonable performance of alternative 44 h enrichment protocols for *Y. enterocolitica*, however this method has not been applied to *Y. pseudotuberculosis* detection. Where molecular detection methods are used (Ferrario et al., 2017; Srinivasan et al., 2017; Li et al., 2018; Liu et al., 2019), the lack of a cultured isolate does not enable further characterisation by subtyping that may be crucial for effective outbreak characterisation and intervention.

Previous studies have shown that ELS analysis allows for concurrent, non-destructive, detection and identification of a range of bacterial foodborne pathogens on solid agar media, and enables the further analysis of the colonies of interest for activities such as subtyping (Banada et al., 2007; Huff et al., 2012; He et al., 2015; Patsekin et al., 2019). We sought to exploit this characteristic to identify *Yersinia* species that may be present in low numbers in foods and amidst a wide range of other bacterial species (Petsios et al., 2016). However, for an ELS-based strategy to be effective, a suitable enrichment method is also needed.

Existing differential isolation strategies include the use of low temperatures, however, these have the disadvantage of extended incubation times (Weagant et al., 2017). Furthermore, although the optimal growth temperature of *Yersinia* species has been described as 28–29°C, they appear to be more rapidly cultured at 37°C under laboratory conditions (Bottone et al., 2005). The relative resistance of *Yersinia* species to alkaline conditions has also been used in conjunction with the development of isolation methods (Weagant et al., 2017; Hallanvuo et al., 2019), yet we found details of alkaline resistance in *Y. pseudotuberculosis* somewhat sparse (Bottone et al., 2005). Therefore, we undertook some preliminary experiments (data not shown) to determine a pH level that appeared to be suited for the recovery of both *Y. enterocolitica* and *Y. pseudotuberculosis* that led us to the specification used in this study. The enrichment broth we selected for use has shown good performance previously (Premaratne et al., 2012), but details of the pH used for *Y. pseudotuberculosis* isolation were not given. The pH we employed is within the range quoted by these authors (Premaratne et al., 2012). Our isolation strategy aimed to recover *Y. enterocolitica* and *Y. pseudotuberculosis* strains in a timely manner, while using alkaline conditions to repress the growth of contaminating flora. Our subsequent use of a nonselective medium enhances the chances of detecting *Yersinia* species, especially since *Y. pseudotuberculosis* grows poorly on Celfsulodin-irgasan-novobiocin (CIN) agar (Fukushima and Gomyoda, 1986; Bosi et al., 1994; Bottone et al., 2005), despite this being a recommended protocol (Weagant et al., 2017). Our results for recovery of spiked autoclaved mince samples supported the efficacy of this approach.

The software used to interpret the ELS profiles derived from individual colonies requires a suitable match to be present in the database to assign a presumptive identity. We considered our prototype database possessed sufficient performance characteristics in terms of separation of the taxa included as assessed by the CV metric (Table 2) for subsequent challenge studies of spiked samples of pork mince with strains of each of *Y. enterocolitica* and *Y. pseudotuberculosis.* Our initial challenge studies using high (10^6^ cfu/g) inocula revealed that 88% of strains presumptively identified by ELS to these species were correctly identified. Confirmation of their identities was attained by subsequent culture purification and DNA analysis using 16S rRNA gene sequence comparisons, a step that could equally as well be done in a clinical laboratory by rapid MALDI-TOF analysis, with results obtained within one working day (Sandalakis et al., 2017). The process still enables further subtyping procedures to be undertaken, should an outbreak be suspected. Our subsequent experiments using lower inoculum sizes demonstrated that most (24/31) isolates identified as *Yersinia* to at least genus level were correctly identified, with operator experience also forming a component. The majority (6/7) of misidentified strains in these experiments represented taxa not initially found in our pork meat studies, and hence not included in our prototype database. The inability to identify organisms not represented in a database is a feature of every identification system, and one we anticipate would be resolved in an updated version of our ELS database that included the missing taxa. Nonetheless, we believe this study clearly indicates the potential value of combining ELS analysis with an effective enrichment and isolation protocol as described here for the expedited screening of foods for yersiniae.

Our observations concerning the ELSA-based separation of *Y. enterocolitica* biovars (Supplementary Table 2) are also intriguing, showing some correlation with earlier studies on serotype discrimination of Shiga-toxigenic *Escherichia coli* strains (Tang et al., 2014). Further studies with additional typed *Y. enterocolitica* strains are warranted to substantiate this observation. Since *Y. pseudotuberculosis* isolates also exhibit considerable serotype variation (Kenyon et al., 2017), such diversity would also be an interesting aspect to explore via ELSA, potentially offering the possibility of isolation, speciation and subtyping concurrently. Furthermore, the inclusion of additional strains enhances the quality of any database, and here may improve resolution of this species from others in the CV matrix (cf. Table 2).

In summary, additional studies are required to further enhance the performance of this approach, and to evaluate its efficacy for other food matrices such as vegetables that have also been implicated as a source of yersiniosis (MacDonald et al., 2016; Nousiainen et al., 2016; Williamson et al., 2016). Such enhancements may be as straightforward as increasing the sample size from 10 g (used here) to 25 g (used routinely: (Weagant et al., 2017). Nonetheless, the prospect of identifying *Y. enterocolitica* and *Y. pseudotuberculosis* contamination in foods within 39 h, compared with 10–21 days using existing conventional approaches is surely an attractive one for improved food safety. Indeed, the fact that we recovered two native *Yersinia* strains with this approach in our studies is a most encouraging sign.

## Data Availability Statement

The original contributions presented in the study are included in the article/Supplementary Material, further inquiries can be directed to the corresponding author/s.

## Author Contributions

SO conceived and supervised the experiments, coordinated inputs, and prepared the manuscript. YZ performed most of the ELS and PCR laboratory work and edited the manuscript. AG provided the essential research materials, undertook some of the ELS work, and edited the manuscript. VP designed the ELS analytical software. VC undertook the laboratory work investigating pH growth range boundaries. SF, HW, and CB provided essential research materials and edited the manuscript. GF, CB, and JL provided essential logistical support and edited the manuscript. JR provided the ELS scanner, essential logistical support, and edited the manuscript. All authors contributed to the article and approved the submitted version.

## Conflict of Interest

The authors declare that the research was conducted in the absence of any commercial or financial relationships that could be construed as a potential conflict of interest.

## References

[B1] Altschul S. F. Gish W. Miller W. Myers E. W. Lipman D. J. (1990). Basic local alignment search tool. *J. Mol. Biol.* 215 403–410. 2231712 10.1016/S0022-2836(05)80360-2

[B2] Bae E. Bai N. Aroonnual A. Bhunia A. K. Hirleman E. D. (2011). Label-Free Identification of Bacterial Microcolonies Via Elastic Scattering. *Biotechnol. Bioengin.* 108 637–644. 10.1002/bit.22980 21246511

[B3] Bae E. Ying D. W. Kramer D. Patsekin V. Rajwa B. Holdman C. (2012). Portable bacterial identification system based on elastic light scatter patterns. *J. Biol. Engin.* 6:12. 10.1186/1754-1611-6-12 PMC3490744 22929757

[B4] Banada P. P. Guo S. L. Bayraktar B. Bae E. Rajwa B. Robinson J. P. (2007). Optical forward-scattering for detection of Listeria monocytogenes and other Listeria species. *Biosens. Bioelectr.* 22 1664–1671. 10.1016/j.bios.2006.07.028 16949268

[B5] Banada P. P. Huff K. Bae E. Rajwa B. Aroonnual A. Bayraktar B. (2009). Label-free detection of multiple bacterial pathogens using light-scattering sensor. *Biosen. Bioelectr.* 24 1685–1692. 10.1016/j.bios.2008.08.053 18945607

[B6] Bancerz-Kisiel A. Pieczywek M. Lada P. Szweda W. (2018). The Most Important Virulence Markers of Yersinia enterocolitica and Their Role during Infection. *Genes* 9:235. 10.3390/genes9050235 29751540 PMC5977175

[B7] Bancerz-Kisiel A. Szweda W. (2015). Yersiniosis - a zoonotic foodborne disease of relevance to public health. *Anna. Agricult. Environ. Med.* 22 397–402. 10.5604/12321966.1167700 26403101

[B8] Batzilla J. Heesemann J. Rakin A. (2011). The pathogenic potential of Yersinia enterocolitica 1A. *Int. J. Med. Microbiol.* 301 556–561. 10.1016/j.ijmm.2011.05.002 21798805

[B9] Bosi E. Madie P. Wilks C. R. (1994). Growth of Yersinia pseudotuberculosis on selective media. *N. Z. Vet. J.* 42:35. 10.1080/00480169.1994.35780 16031741

[B10] Bottone E. J. Bercovier H. Mollaret H. H. (2005). Genus XLI. Yersinia. *Bergey’s Manu. Syst. Bacteriol.* 2 838–848.

[B11] CDC. (2019). *Yersinia enterocolitica.* Available at: https://www.cdc.gov/yersinia/index.html (accessed August 28, 2020).

[B12] ECDC. (2019). *Yersiniosis*. Annual epidemiological report for 2018. (Stockholm: ECDC).

[B13] Ferrario C. Lugli G. A. Ossiprandi M. C. Turroni F. Milani C. Duranti S. (2017). Next generation sequencing-based multigene panel for high throughput detection of food-borne pathogens. *Int. J. Food Microbiol.* 256 20–29. 10.1016/j.ijfoodmicro.2017.05.001 28578266

[B14] Fois F. Piras F. Torpdahl M. Mazza R. Ladu D. Consolati S. G. (2018). Prevalence, bioserotyping and antibiotic resistance of pathogenic Yersinia enterocolitica detected in pigs at slaughter in Sardinia. *Int. J. Food Microbiol.* 283 1–6. 10.1016/j.ijfoodmicro.2018.06.010 29929063

[B15] Fukushima H. Gomyoda M. (1986). Growth of Yersinia pseudotuberculosis and Yersinia enterocolitica biotype 3B serotype O3 inhibited on cefsulodin-Irgasan-novobiocin agar. *J. Clin. Microbiol.* 24 116–120. 10.1128/jcm.24.1.116-120.1986 3722357 PMC268844

[B16] Hallanvuo S. Herranen M. Jaakkonen A. Nummela M. Ranta J. Botteldoornl N. (2019). Validation of EN ISO method 10273-Detection of pathogenic Yersinia enterocolitica in foods. *Int. J. Food Microbiol.* 288 66–74. 10.1016/j.ijfoodmicro.2018.01.009 29395387

[B17] He Y. P. Reed S. Bhunia A. K. Gehring A. Nguyen L. H. Irwin P. L. (2015). Rapid identification and classification of Campylobacter spp. using laser optical scattering technology. *Food Microbiol.* 47 28–35. 10.1016/j.fm.2014.11.004 25583335

[B18] Huff K. Aroonnual A. Littlejohn A. E. F. Rajwa B. Bae E. Banada P. P. (2012). Light-scattering sensor for real-time identification of Vibrio parahaemolyticus, Vibrio vulnificus and *Vibrio cholerae* colonies on solid agar plate. *Microbial. Biotechnol.* 5 607–620. 10.1111/j.1751-7915.2012.00349.x 22613192 PMC3815873

[B19] Joutsen S. Laukkanen-Ninios R. Henttonen H. Niemimaa J. Voutilainen L. Kallio E. R. (2017). Yersinia spp. in Wild Rodents and Shrews in Finland. *Vector Borne Zoonotic Dis.* 17 303–311. 10.1089/vbz.2016.2025 28332937

[B20] Kenyon J. J. Cunneen M. M. Reeves P. R. (2017). Genetics and evolution of Yersinia pseudotuberculosis O-specific polysaccharides, a novel pattern of O-antigen diversity. *FEMS Microbiol. Rev.* 41 200–217. 10.1093/femsre/fux002 28364730 PMC5399914

[B21] Le Guern A. S. Martin L. Savin C. Carniel E. (2016). Yersiniosis in France, overview and potential sources of infection. *Int. J. Infect. Dis.* 46 1–7. 10.1016/j.ijid.2016.03.008 26987478

[B22] Li Y. R. Su H. W. Lan Y. J. (2018). Simultaneous Detection of Yersinia enterocolitica and Listeria monocytogenes in Foodstuffs by Capillary Electrophoresis and Microchip Capillary Electrophoresis Laser-Induced Fluorescence Detector. *J. Aoac Int.* 101 1833–1838. 10.5740/jaoacint.17-0507 29843867

[B23] Liu Y. Gao Y. Wang T. Dong Q. G. Li J. W. Niu C. (2019). Detection of 12 Common Food-Borne Bacterial Pathogens by TaqMan Real-Time PCR Using a Single Set of Reaction Conditions. *Front. Microbiol.* 10:222. 10.3389/fmicb.2019.00222 PMC6381072 30814987

[B24] MacDonald E. Einoder-Moreno M. Borgen K. Brandal L. T. Diab L. Fossli O. (2016). National outbreak of Yersinia enterocolitica infections in military and civilian populations associated with consumption of mixed salad. *Norway 2014*. *Eurosurveillance* 21 11–19. 10.2807/1560-7917.ES.2016.21.34.30321 PMC5144932 27588690

[B25] Nousiainen L. L. Joutsen S. Lunden J. Hanninen M. L. Fredriksson-Ahomaa M. (2016). Bacterial quality and safety of packaged fresh leafy vegetables at the retail level in Finland. *Int. J. Food Microbiol.* 232 73–79. 10.1016/j.ijfoodmicro.2016.05.020 27257744

[B26] Patsekin V. On S. Sturgis J. Bae E. Rajwa B. Patsekin A. (2019). “Classification of *Arcobacter* species using variational autoencoders,” in *SPIE 11016, Sensing for Agriculture and Food Quality and Safety XI*, Vol. 1101608 (Washington: SPIE).

[B27] Pattis I. Cressey P. Lopez L. Horn B. Soboleva T. (2019). *Annual Report Concerning Foodborne Disease in New Zealand 2018.* New Zealand: Ministry for Primary Industries.

[B28] Petsios S. Fredriksson-Ahomaa M. Sakkas H. Papadopoulou C. (2016). Conventional and molecular methods used in the detection and subtyping of Yersinia enterocolitica in food. *Int. J. Food Microbiol.* 237 55–72. 10.1016/j.ijfoodmicro.2016.08.015 27543816

[B29] Premaratne A. Wilson T. King N. Hudson J. A. (2012). Growth of Yersinia enterocolitica and Y. pseudotuberculosis in Yersinia selective enrichment broth according to Ossmer. *J. Microbiol. Methods* 89 198–200. 10.1016/j.mimet.2012.03.008 22450139

[B30] Rosner B. M. Werber D. Hohle M. Stark K. (2013). Clinical aspects and self-reported symptoms of sequelae of Yersinia enterocolitica infections in a population-based study, Germany 2009-2010. *BMC Infect. Dis.* 13:236. 10.1186/1471-2334-13-236 PMC3669037 23701958

[B31] Sandalakis V. Goniotakis I. Vranakis I. Chochlakis D. Psaroulaki A. (2017). Use of MALDI-TOF mass spectrometry in the battle against bacterial infectious diseases, recent achievements and future perspectives. *Exp. Rev. Proteom.* 14 253–267. 10.1080/14789450.2017.1282825 28092721

[B32] Srinivasan V. Stedtfeld R. D. Tourlousse D. M. Baushke S. W. Xin Y. Miller S. M. (2017). Diagnostic microarray for 14 water and foodborne pathogens using a flatbed scanner. *J. Microbiol. Methods* 139 15–21. 10.1016/j.mimet.2017.04.009 28438642 PMC5491306

[B33] Tang Y. J. Kim H. Singh A. K. Aroonnual A. Bae E. Rajwa B. (2014). Light Scattering Sensor for Direct Identification of Colonies of *Escherichia coli* Serogroups O26, O45, O103, O111, O121, O145 and O157. *PLoS One* 9:e105272. 10.1371/journal.pone.0105272 25136836 PMC4138183

[B34] Van Damme I. De Zutter L. Jacxsens L. Nauta M. J. (2017). Control of human pathogenic Yersinia enterocolitica in minced meat, Comparative analysis of different interventions using a risk assessment approach. *Food Microbiol.* 64 83–95. 10.1016/j.fm.2016.12.006 28213039

[B35] Weagant S. D. Feng P. Stanfield J. T. (2017). *BAM Chapter 8, Yersinia enterocolitica. Bacteriological Analytical Manual (BAM) US Food and Drug Administration.* Silver Spring: US Food and Drug Administration.

[B36] Weisburg W. G. Barns S. M. Pelletier D. A. Lane D. J. (1991). 16S ribosomal DNA amplification for phylogenetic study. *J. Bacteriol.* 173 697–703. 10.1128/jb.173.2.697-703.1991 1987160 PMC207061

[B37] Williamson D. A. Baines S. L. Carter G. P. da Silva A. G. Ren X. Y. Sherwood J. (2016). Genomic Insights into a Sustained National Outbreak of Yersinia pseudotuberculosis. *Genome Biol. Evolut.* 8 3806–3814. 10.1093/gbe/evw285 PMC5521734 28173076

