# Supplementary material for: Elastic Light Scatter Pattern Analysis for the Expedited Detection of Yersinia Species in Pork Mince: Proof of Concept
Source: Front Microbiol. 2021 Feb 17;12:641801. doi: 10.3389/fmicb.2021.641801 (PMC7928378; doi:10.3389/fmicb.2021.641801)
Supplement: Supplementary file 1 [file Data_Sheet_1.pdf]

SUPPLEMENTARY DATA: **Elastic Light Scatter pattern analysis for the expedited detection of *Yersinia* species in pork mince: proof of concept**

Stephen L. W. On[1*], Yuwei Zhang[1], Andrew Gehring[2], Valery Patsekin[3], Venkata Chelikani[1], Steve Flint[4], Haoran Wang[4], Craig Billington[5], Graham C. Fletcher[6], James Lindsay[7] and J. Paul Robinson[8]

[1] Dept. Wine, Food & Molecular Biosciences, Lincoln University, New Zealand; [2] Eastern Regional Research Center, Agricultural Research Service, USDA , Wyndmoor, Pennsylvania; [3] Dept. Basic Medical Science, Purdue University, W. Lafayette, USA; [4] Massey University, Palmerston North, New Zealand; [5] Institute of Environmental Science and Reseach, Christchurch, New Zealand; [6] New Zealand Institute for Plant & Food Research Limited, Auckland, New Zealand; [7] Agricultural Research Service, Office of National Programs, USDA, Washington; [8] Weldon School of Biomedical Engineering, Purdue University, W. Lafayette, USA.

Supplementary Table 1. CV matrix for individual *Yersinia* species examined in initial test database.

	Y. enterocolitica	*Y. pseudotuberculosis*	*Y. frederiksenii*	*Y. intermedia*	*Y. kristensenii*
Y. enterocolitica	95.6	0.5	2.2	1.2	0.5
Y. pseudotuberculosis	1.8	90.4	0	0.1	7.7
Y. frederiksenii	20.8	0	74.8	1.5	2.8
Y. intermedia	51.4	0	15.3	30.7	2.6
Y. kristensenii	1.1	3.8	5.5	0.4	89.3

Supplementary Table 2. CV matrix for *Y. enterocolitica* biotypes examined.

	Y. enterocolitica biotype 1A	*Y. enterocolitica* biotype 1B	*Y. enterocolitica* biotype 2	*Y. enterocolitica* biotype 3	*Y. enterocolitica* biotype 4
Y. enterocolitica biotype 1A	90.8	0.1	8.8	0.3	0
Y. enterocolitica biotype 1B	0	92.3	1.5	2.6	3.7
Y. enterocolitica biotype 2	1.1	0.7	94.4	0.9	2.8
Y. enterocolitica biotype 3	1.6	4	2.1	92.3	0
Y. enterocolitica biotype 4	0.1	0.7	6.9	0	92.3
